# Outcomes in cardiac surgery in 500 consecutive Jehovah's Witness patients: 21 year Experience

**DOI:** 10.1186/1749-8090-7-95

**Published:** 2012-09-27

**Authors:** Claude D Vaislic, Nicolas Dalibon, Oliver Ponzio, Maguette Ba, Eric Jugan, Franck Lagneau, Philippe Abbas, Yves Olliver, Didier Gaillard, Francois Baget, Michel Sportiche, Antoine Chedid, Georges Chaoul, Philippe Maribas, Christiane Dupuy, Bruno Robine, Nicolas Kasanin, Herve Michon, Jean-Michel Ruat, Michel Habis, Touhami Bouharaoua

**Affiliations:** 1 CHP PARLY 2, 21 Rue Moxouris, Le Chesnay, 78150, France

## Abstract

**Background:**

Refusal of heterogenic blood products can be for religious reasons as in Jehovah's Witnesses or otherwise or as requested by an increasing number of patients. Furthermore blood reserves are under continuous demand with increasing costs. Therefore, transfusion avoidance strategies are desirable. We describe a historic comparison and current results of blood saving protocols in Jehovah's Witnesses patients.

**Methods:**

Data on 250 Jehovah's Witness patients operated upon between 1991 and 2003 (group A) were reviewed and compared with a second population of 250 patients treated from 2003 to 2012 (group B).

**Results:**

In group A, mean age was 51 years of age compared to 68 years in group B. An iterative procedure was performed in 13% of patients in group B. Thirty days mortality was 3% in group A and 1% in group B despite greater operative risk factors, with more redo, and lower ejection fraction in group B. Several factors contributed to the low morbidity-mortality in group B, namely: preoperative erythropoietin to attain a minimal hemoglobin value of 14 g/dl, warm blood cardioplegia, the implementation of the Cornell University protocol and fast track extubation.

**Conclusions:**

Cardiac surgery without transfusion in high-risk patients such as Jehovah Witnesses can be carried out with results equivalent to those of low risk patients. Recent advances in surgical techniques and blood conservation protocols are main contributing factors.

## Background

Patients undergoing cardiac surgery receive blood transfusion in 20-80% of cases. Certain studies suggest that half of these transfusions are unnecessary and can be avoided [1,2]. Furthermore, multivariate analyses have identified blood transfusion as an independent factor for mortality in the long term. Patients and their kin, informed of the feasibility of surgery without blood transfusion have frequently opted for this choice. On the other hand the need for blood products is on the rise due to the aging demographic of the population, a reduction in the number of potential donors and an increase in the costs of transfusions due to increased vigilance over the quality of blood and more stringent sanitary measures [3,4].

Over the course of the last 21 years, we have performed cardiac surgery on 500 patients of the Jehovah witness faith which requires its followers to refrain from blood transfusions. In all of these cases, no transfusion was performed. Over this period, the techniques of blood preservation have improved greatly and have become rather systematized, together with an overall improvement in the operative risk of cardiac surgery. In order to measure the effects of advances in blood conservation techniques, we compared our first 250 Jehovah Witness cases to a subsequent group of 250 patients, paying particular attention to the impact of the introduction of preoperative erythropoietin (EPO) in the latter group.

## Methods

Between January 1991 and January 2012 , elective cardiac surgery was performed on 500 Jehovah’s Witness patients, all of whom gave informed consent with emphasis on the risks of non-transfusion. During this period, 622 patients were referred to our team and 122 were excluded (Table 1). Until 1998, 37 patients were excluded because their hemoglobin level was below 14 g/l, which was no longer the case afterwards due to the systematic use of Erythropoietin. Consultation with local ethics committee established inclusion and exclusion criteria, thus we excluded emergency procedures, congenital heart disease, cardiogenic shock, patients in whom a prolonged EPO treatment did not produce a hemoglobin level of 14 g, patients with problems of hemostasis or those in whom coagulopathy was anticipated following the implementation of extra-corporal circulation (cold agglutinin coagulopathy, sickle cell disease) and patients with thoraco-abdominal aneurysms in which we could not reasonably avoid transfusion. Three patients suffering from aortic dissection were excluded due to initial preoperative hemoglobin levels below 15 g/l. Three patients could not be operated upon due to it being impossible to attain sufficient levels of hemoglobin prior to the scheduled procedure, despite a month of EPO treatment. One of these patients showed signs of an increase in the size of a type 1 aortic dissection which became chronic following his refusal of a transfusion despite the advice of the care team. The other patient could not be operated upon due to a reaction to EPO of unidentifiable cause.

**Table 1:** Exclusion Criteria

	**n**
Hemoglobin < 14 g/l prior to 1998	37
Emergency Procedures	36
Complex Congenital Heart Disease	5
Cardiogenic Shock	10
Ineffective EPO therapy	3
Sickle cell disease	1
Non correctable hemostasis abnormality (low platelet count)	
Body Mass Index < 15	7
Thoraco-abdominal aneurysm	16

The method of blood conservation utilized over the years has evolved, resulting in the adoption of the “Comprehensive Multimodality Blood Conservation Program” developed by Cornell University, detailed on Table 2[5,6]. Aprotinin and EPO were used as dictated by protocol (Table 3). Aprotinin, which enhances erythrocyte hematopoiesis was used in all our patients according to Hammersmith Hospital protocol until its withdrawal in 2007 , then aminocaproic acid is used in all cases . Prior to 1998, all patients with hemoglobin levels below 14 g/l were refused. After 1998, EPO was used systematically in all our patients with hemoglobin levels below 14 g/l prior to surgical intervention. The issue of cost did not arise as a factor as this medication was paid for by the patients. The relatively high level of hemoglobin was intended to extend the safety margin in these patients in whom postoperative transfusion was not an option.

**Table 2:** Transfusion Related Risk Factors

**Variable**	**Group A n =** **250**	**Group B n =** **250**	***P***
	**1991-1998**	**1998 - 2012**	
Blood Volume (ml)	5 200 +/- 480	5000 +/- 610	>0.05
Preoperative HCT (%)	41+/-2	40+/-4	<0.001
Preoperative erythrocyte mass (ml)	2215+/- 250	2010+/-200	< 0.05
Platelets	220 ± 60	180 ± 50	< 0 .05
Body Mass Index	25 ± 5	24 ± 3	>0.05
Age	51+/-7	68+/-5	<0.001
Female gender	11	28	<0.001
Diabetes	24	31	>0.05
Renal Failure	10%	10%	>0.05
Euroscore	4.1 +/- 0.8	4.8 +/- 1.3	<0.05
Ejection Fraction	0.45+/-0.2	0.40+/-0.1	<0.001
Aortic Clamp Time	70+/-15	65+/-30	< 0.05
Ischemic Time	55+/-10	48+/-15	>0.05
Blood Loss (ml)	515 ± 310	350 ± 140	<0.001

**Table 3:** Protocols

	
1). Protocol for blood conservation	**a) Preoperative**
	EPO if hemoglobin levels are <14 g/l
	Iron in all cases
	Limit the removal of blood in frequency and quantity (use pediatric tubes)
	Aprotinin (Hammersmith half-protocol)
	Avoid hematomas following angiography and PCI.
	**b) Perioperative**
	Retropriming
	Short CPB circuit. Heparinization 3 mg/kg body weight reversed by equivalent dose of protamine IV
	CPB conducted in normothermia (minimal temperature drift 36 °C)
	Warm cardioplegia
	Cell Saver
	Minimally invasive surgical techniques
	Meticulous closure
	**c) Post operative**
	Reduce blood retrieval in frequency and quantity (use pediatric tubes)
	Reoperate if blood loss continues at 100 cc for three hrs, or immediately if > 200 cc in one hour.
	EPO if hematocrit < 24% at time of reoperation
2). Protocol of Aprotonin Administration (Hammersmith Half-Protocol) until its withdrawal in 2007 , then aminocaproic acid is used in all cases	1 million KIU (140 mg) IV at induction of anesthesia, 1 million KIU (140 mg) at completion of CPB and 250,000 KIU (35 mg) IV per hour until skin closure or until a maximum dose of 1 million KIU.
3). Protocol of Erythropoietin Administration	300 UI/Kg IV + 500 UI/Kg subcutaneously on admission followed by 500 UI/Kg subcutaneously every second day.
	+ Iron 325 mg PO 3 times a day
4). Retropriming	Avoid hemodilution during priming of CPB by passive drainage of blood from venous system
5). MiniCPB	CPB using centrifugal pump with small volume , in a closed circuit

Elective cardiac catherization was done a few weeks before surgery to avoid drops in hb and recently use of a vascular closure device has been systematized.

As preoperative antiplatelet therapy is an identified risk factor of bleeding requiring excess transfusion , we discontinued clopidogrel and aspirin in all cases prior to surgery (35 patients in group B). Aspirin alone was discontinued as well. 15 patients in group B had drug eluting stents implanted and surgery was postponed until three months later in order to be able to effectively discontinue all antiplatelet agent according to our protocol.

In January 2000, our therapeutic procedures for coronary and valve surgery were enriched by the implementation of mini-extracorporal circulation with retropriming. This consists of a centrifugal pump in a closed circuit. The circuit is filled with the blood of the patient which is drained passively through a venous canula, thus eliminating the crystalloid solutions which refill the circuit and result in hemodilution.

We compared the operative risk, as measured by Euroscore, as well as mortality, morbidity, hemoglobin levels and blood loss in the first 250 cases, operated upon prior to 2003 (group A), to 250 patients operated upon thereafter (group B). Moreover, due to the fact that the patients in group B were at greater risk than those in group A, we also verified that we were able to obtain results which were nearly equivalent. The first objective was to compare the mortality rates of the first 30 days following operation and the incidence of postoperative blood loss in the two groups. The second objective was to compare the clinical evolution and hemoglobin levels throughout the hospital stay.

### Statistical Analysis

The hypothesized normality of the distribution of continuous variables was verified by a Kolmogorov-Smirnoff test. The continuous variables are expressed as means of standard deviation and the differences were evaluated by Student *T* test. Variable analysis was conducted by Chi-Square and Fisher tests according to the size of the sample. Statistical significance was assumed when the P value was found to be less than 0.05. Stataxt software packages (Cytel Software Corporation, Cambridge, Mass) were used in the statistical analysis.

## Results

The type of operation, the mortality and the morbidity in the first postoperative 30 days are shown in Tables 4 and 5. Significant technical developments and transfusion-related risk factors are shown in Tables 6 and 2.

**Table 4:** **Type of intervention from January 1991 to October 2003 **

	**GROUP A**		**GROUP B**	
	**1991-2003**		**2003-2012**	
Intervention		*30 days Mortality*		*30 days Mortality*
Aortic Valve Replacement	102	1	63 (4 reoperations)	1
			(5 hybrids )*	
Mitral Valve Replacement	5		9	
Double Valve Replacement	1		6	
Mitral Valve-repair	1		21	
CABG	140	2	146 (9 reoperations)	
			(25 hybrids)*	
Aortic Dissection	0		2	
ASD repair	1			
CIA + SINUS VENOSUS			1	
VSD Repair LV Rupture			1	
			1	

*ASD* (atrial septal defect), *CABG* (coronary artery bypass grafting), *VSD* (ventricular septal defect), *Hybrids* : coronary revascularization associating surgery and angioplasty.

**Table 5:** Results

**RESULTS**	**Group A**	**Group B**	***P***
30 Day Mortality	3	1	>0.05
Reoperation following bleeding	4	3	>0.05
Acute MI (tropoponine + CPK MB)	2	1	>0.05
Mediastinitis	0	0	>0.05
Stroke	1	0	>0.05
Renal Failure (creatinine level > 2 mg/dL)	18	14	>0.05
Atrial Fibrillation	20	24	>0.05
Ultra-fast Track	0	77	< 0.001
ICU stay (Days)	3 +/-1	4 +/-1	>0.05
Mechanical Ventilation (hrs)	8 +/- 4	2 +/- 1	< 0.001
Hospitalization >7 days	32	18	< 0.001

CPK (creatinin phosphokinase), ICU (intensive care unit).

**Table 6:** Methods of Blood Conservation

**Procedure**	**Group A n =** **250**	**Group B n =** **250**	***P***
	**1998-2003**	**2003-2012**	
Retro-priming	0	250	<0.001
Erythropoietin	0	203	<0.001
Extra-corporal mini-circulation	0	152	<0.001

None of these patients received transfusions regardless of the circumstances of the procedure. All benefited from treatment equivalent to what they would have received had they not been Jehovah’s witnesses. This is particularly true for the choice of valve substitute or conduit graft in revascularization procedures which were again not influenced by the fact that they were Jehovah’s witnesses.

The operative risk of these patients was assessed by a passable Euroscore of 4.1 ± 0.8 to 4.9 ± 1.3 (p < 0.05) in groups A and B respectively. In group B, we took into account two aortic dissections and one adult congenital heart disease, as well as patients who had previously underwent cardiac surgery. In group B, although the proportion of patients who were diabetic, female, or had low ejection fractions increased, and the number of patients over 70 years of age doubled, the incidence of massive bleeding (defined as leading to hypovolemia or shock) decreased.

**Figure 1:**
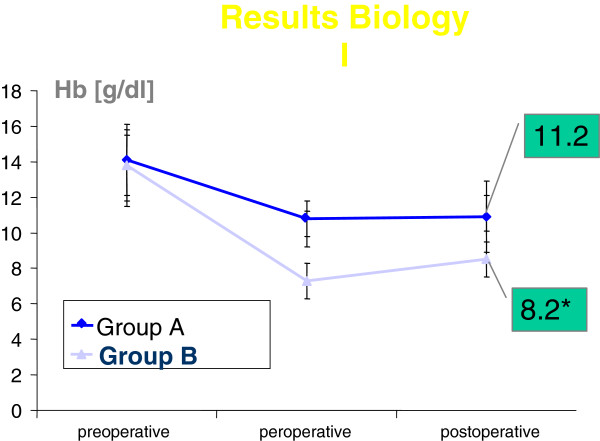
** Additional file ****1 ****is the HB comparison levels.** Preo means preoperative. Posto means postoperative. Mi means minimum during bypass.

Despite the increased risk associated with group B, operative mortality diminished, due to progress in surgical and medical techniques. We did not observe significant renal failure, mediastinitis or enzymatic increase indicative of myocardial suffering. Conversely we did not observe stents occlusion in the patients with drug eluting stents in whom antiplatelet therapy has been discontinued. Thus, recently-operated patients had a greater level of hemoglobin. The thoracic drain output was halved (515 ml ± 31 vs 350 ml ± 140, in groups A and B respectively) without major modification or a more minimally invasive surgical technique, as shown by the equivalent ischemic times. The perioperative hemoglobin and hematocrit levels were better controlled in group B (Figures 1 and 2). 223/250 of the patients in group B were treated with preoperative EPO due to insufficient hemoglobin levels (11 ± 2 g/l).

The four deaths were due to an ulceration of the gastro-duodenal artery which could not be treated by endoscopic sclerotherapy, renal failure and two hemorrhages which occurred at the site of operation. Any bleeding greater than 200 ml within the first 3 hours following surgery was an indication for re-exploration which occurred in 4 patients in group A and 3 in group B.

## Discussion

1) Transfusion pro and con

Anaemia is a potent risk factor for mortality and morbidity in surgical patients, and its management has begun to shift away from allogeneic blood transfusion in recent years.

Adverse effects of anaemia have been demonstrated specifically in the perioperative setting. A preoperative hemoglobin concentration of less than 6 g/dL increases the risk of death 30 days after surgery by a factor of 26 relative to a concentration of 12 g/dL or greater in surgical patients who declined blood transfusion for religious reasons [7].

The safety of the blood supply has improved. Sophisticated testing has led to a decline in the risk of transfusion-related transmission of HIV, the hepatitis C virus, and the hepatitis B virus.

But there is a continual rise in fatal TRALI (transfusion-related acute lung injury) cases in the United States from 2001 to 2006. TRALI occurred in more than half of all transfusion-related fatalities reported to the FDA in 2006, a higher number than for any other single cause [8].

At the same time, there is evidence that hemovigilance can reduce the risk of TRALI. TRALI accounted for 6.8% of all transfusion-related adverse events reported in the United Kingdom during the period 1996–2003. This proportion declined to just 1.9% in 2006 [9].

Finally, despite the progress in screening blood for HIV and the hepatitis viruses, some additional infections now must be considered when assessing blood supply safety. These include diseases newly recognized as being transmissible by blood, for which blood donor screening is not currently available, or that are newly emergent infections for which the potential for spread by transfusion is unknown.

The Transfusion Requirement in Critical Care (TRICC) trial was conducted in 838 critically ill patients in intensive care settings. Patients were randomized to a strategy of either liberal transfusion (which begun when hemoglobin fell below 10 g/dL) or restrictive transfusion (which begun when hemoglobin fell below 7 g/dL). The restrictive strategy was associated with significantly lower mortality in two subgroups: patients with myocardial infarction and patients with pulmonary edema.

Rao et al. performed a meta-analysis of three large international trials of patients with acute coronary syndromes to determine whether blood transfusion to correct anaemia in this setting was associated with improved survival [10]. They found significantly higher mortality among patients who underwent transfusion compared with those who did not, prompting them to urge caution in the use of transfusion to maintain arbitrary hematocrit levels in stable patients with ischemic heart disease in an observational cohort study of 11,963 patients who underwent isolated coronary artery bypass graft surgery, each unit of red blood cells transfused was associated with an incrementally increased risk of adverse outcome (eg, mortality, renal injury, need for ventilator support, lengthened hospital stay, infection) [11]. The latter study found that transfusion was the single factor most reliably associated with increased risk of postoperative morbidity

2) Techniques of Blood Conservation

In 2005 we reported our initial experience in 200 Jehovah’s witness patients undergoing cardiac surgery [12]. Since then , we have had to deal with the withdrawal of Aprotinin which we thought was an important weapon in our therapeutic armentarium. The change of protocol by 1 g at induction and 1 g at the end of bypass of EACA did not alter our final results and this change did not alter in our bleeding control in any way. Fifty years of research into the effectiveness and safety of prophylactic use of antifibrinolytics for cardiac surgical patients demonstrate that aprotinin and EACA reduce postoperative blood loss and consequently , the requirement for blood transfusions and reoperation for bleeding compared to placebo or treatment. Traditionally , evidence in favor of EACA is not compelling , but in head-to-head comparisons there is an increased risk of death among patients receiving aprotinin compared to those on lysine analog as indicated by the BART trial. All of these have been demonstrated on huge series including over 12,000 patients. These differences did not appear in our two small groups. The aim of all efforts to reduce the need of allogeneic blood transfusions is to avoid associated risks. There should particularly be a favourable effect according to the rate of transfusion-transmitted virus infections and immunological side-effects. The acceptance of an individually adjusted lowest haematocrit level and the minimisation of intra-operative blood loss by the application of optimal surgical techniques are among the most essential strategies to reduce or even avoid allogeneic blood transfusions

Actively reducing the number of patients requiring blood transfusion after cardiac surgery is a process which involves actors at every stage of hospitalization and, as such, requires more than the simple minimization of surgical invasiveness once held to be a key factor [13-15] each detail in the treatment of these patients is crucial. Starting preoperatively by raising the level of hemoglobin by use of EPO and iron therapy, eliminating any hemodilution, to proper operative field drainage to reduce the incidence of hematomas related to various invasive maneuvers over the course of hospitalization, keeping the hemoglobin value under 15.5 to avoid associated complications with high hematocrit value [16]. The most recent example of this comprehensive multimodality approach is the mini-cardiopulmonary bypass system that we have been using for the last three years, combined with retro-priming and a cell saver [16-21] which maintains a constant hemoglobin level, often low in cardiac patients. Volume expansion in these patients is associated with a real risk of hemodilution and active participation of the anesthetist is essential to avoid additional iatrogenic vasoplegia, which is greatly facilitated by concentration-aimed ultra-fast-track anesthesia protocols [22]. However, multifactorial progress has been made since the series published by Cooley [14], which has recently been confirmed by Jassar [13]. In contrast with teams who use systemic hypothermia or crystalloid cardioplegia, normothermic surgery provides the same rheological results without hemodilution. The complete, unmodified Cornell University protocol has therefore been systematically employed [6]. This attitude is all the more important in that preoperative hemodilution induced by deferred auto-transfusion cannot be performed upon Jehovah’s Witnesses who refuse any interruption of the blood circulation for religious reasons.

3) Legal and Ethical Aspects of Non Transfusion

It has been suggested that patients who receive transfusions following cardiac surgery had twice the long-term mortality risk than patients who did not receive transfusions [4,15]. The administration of blood transfusions therefore appears to be an independent factor of death. If these results attain factual status, then for both medical and legal reasons, all efforts must be made to avoid recourse to blood transfusion not only amongst Jehovah’s Witnesses, but in all patients. A review of the existing literature on methods of blood transfusion indicates not only a discordance in practice amongst medical teams, but even amongst members of the same team: yet the morbidity-mortality rates are ubiquitous in the first 30 postoperative days [1,2,23]. Therefore, it appears desirable to replace these highly unsystematic methods of transfusion with a protocol of blood conservation which involves all members of the caring team. To undertake such a protocol is both medically justified and feasible.

The restraining factor in the implementation of such a protocol is cost, as the proposed blood conservation protocol relies upon two medications: aprotinin and EPO [3,24]. Hence, the cost-benefit analysis depends primarily on the price of EPO. It must be remembered that EPO takes effect with some delay, its effectiveness is uncertain and, at present, its cost is prohibitively expensive. The extent of the delay in its effectiveness depends on the degree of preoperative anaemia. In France, EPO is used in the treatment of anaemia linked to renal failure, and occasionally preoperatively in anaemic patients.

The decision not to administer blood transfusions presents the medical team with an ethical dilemma as, in extreme circumstances, the members may be confronted with a situation in which a patient who could otherwise been saved must be left to die in order for their choice of non transfusion to be respected. Not only does this present an ethical dilemma for doctors but, in France, also presents a potential legal dilemma, since the preoperative consent form signed by the patient does not hold value, in light of the principal of “*non-assistance to a personin danger*”, which is in direct opposition to the voiced desires of the patient. It is thus held to be of the utmost importance to reduce all possible risks related to non transfusion [23] in order to prevent the possibility of extreme situations in which such ethical and legal dilemmas may arise. We succeeded in this aspect in all cases except four who were conscious and repeatedly refused transfusion despite the propositions of the attending medical professionals. These preoperative exclusions account for the better results obtained in group B compared with the outcomes attained in group A. This rigorous selection process allowed us to propose standard techniques of treatment to all our patients, such as mitral valve repair and total arterial revascularization, a technique which enhances blood conservation. Redo operations did not present a problem as we systematically applied the technique described by O’Bryan which has not resulted in a single major cardiac event in over 300 re-operations [20]. Nevertheless, these operative redos remain risky endeavors which must be evaluated on a case by case basis.

## Conclusion

Cardiac surgery without transfusion may be performed with an equivalent risk to standard surgery. This has been made possible by the progress in methods of blood conservation employing pharmacological agents - namely aprotinin then EACA - and erythropoietin and improving the physical design of cardio-pulmonary bypass techniques. Nevertheless, patient selection remains extremely important to achieve these results.

## Competing interests

The authors declare that they have no competing interests.

## Authors’ contribution

CV carried out the statistics study. All authors read and approved the final manuscript.

## Supplementary Material

Additional file 1 Results of biology. Click here for file
