# Supplementary material for: Outcomes in cardiac surgery in 500 consecutive Jehovah's Witness patients: 21 year Experience
Source: J Cardiothorac Surg. 2012 Sep 27;7:95. doi: 10.1186/1749-8090-7-95 (PMC3487917; doi:10.1186/1749-8090-7-95)
Supplement: Additional file 1 — Results of biology. [file 1749-8090-7-95-S1.docx]

preoperative

postoperative

peroperative

**Hb [g/dl]**

8.2*

11.2

**Group B**

Group A

18

16

14

12

10

8

6

4

2

0

**Results Biology I**

**Hb**
